# Optimal nutrition during the period of mechanical ventilation decreases mortality in critically ill, long-term acute female patients: a prospective observational cohort study

**DOI:** 10.1186/cc7993

**Published:** 2009-08-11

**Authors:** Rob JM Strack van Schijndel, Peter JM Weijs, Rixt H Koopmans, Hans P Sauerwein, Albertus Beishuizen, Armand RJ Girbes

**Affiliations:** 1 Department of Intensive Care Medicine, VU University Medical Centre, PO Box 7057, 1007 MB Amsterdam, the Netherlands; 2 Department of Nutrition and Dietetics, VU University Medical Centre, PO Box 7057, 1007 MB Amsterdam, the Netherlands; 3 Palmgracht 44 B, 1015 HN Amsterdam, the Netherlands

## Abstract

**Introduction:**

Optimal nutrition for intensive care patients has been proposed to be the provision of energy as determined by indirect calorimetry, and protein provision of at least 1.2 g/kg pre-admission weight per day. The evidence supporting these nutritional goals is based on surrogate outcomes and is not yet substantiated by patient oriented, clinically meaningful endpoints. In the present study we evaluated the effects of achieving optimal nutrition in ICU patients during their period of mechanical ventilation on mortality.

**Methods:**

This was a prospective observational cohort study in a mixed medical-surgical, 28-bed ICU in an academic hospital. 243 sequential mixed medical-surgical patients were enrolled on day 3–5 after admission if they had an expected stay of at least another 5–7 days. They underwent indirect calorimetry as part of routine care. Nutrition was guided by the result of indirect calorimetry and we aimed to provide at least 1.2 g of protein/kg/day. Cumulative balances were calculated for the period of mechanical ventilation. Outcome parameters were ICU, 28-day and hospital mortality.

**Results:**

In women, when corrected for weight, height, Apache II score, diagnosis category, and hyperglycaemic index, patients who reached their nutritional goals compared to those who did not, showed a hazard ratio (HR) of 0.199 for ICU mortality (CI 0.048–0.831; *P *= 0.027), a HR of 0.079 for 28 day mortality (CI 0.013–0.467; *P *= 0.005) and a HR of 0.328 for hospital mortality (CI 0.113–0.952; *P *= 0.04). Achievement of energy goals whilst not reaching protein goals, did not affect ICU mortality; the HR for 28 day mortality was 0.120 (CI 0.027–0.528; *P *= 0.005) and 0.318 for hospital mortality (CI 0.107–0.945; *P *= 0.039). No difference in outcome related to optimal feeding was found for men.

**Conclusions:**

Optimal nutritional therapy improves ICU, 28-day and hospital survival in female ICU patients. Female patients reaching both energy and protein goals have better outcomes than those reaching only the energy goal. In the present study men did not benefit from optimal nutrition.

## Introduction

Nutrition is an integral and important part of therapy in the ICU. Nutritional therapy aims at conservation or restoration of the body protein mass and of provision of adequate amounts of energy. On a hypothetical basis, surrogate markers for optimal nutrition with regard to energy and protein provision have proposed to be the delivery of energy as measured by indirect calorimetry, and provision of 1.2 to 1.5 g of protein per kg of pre-admission weight for critically ill patients [1]. It has been shown that inadequate provision of energy correlates with the occurrence of complications, such as adult respiratory distress syndrome, infections, renal failure, pressure sores and need for surgery [2,3]. Recently, Anbar and colleagues [4] have provided preliminary evidence in a group of 50 patients with an expected ICU stay of more than three days, that provision of energy according to indirect calorimetry led to cumulative positive energy balances whereas the control group (targeted at 25 kcal/kg) had negative cumulative energy balances; hospital morbidity and hospital mortality decreased in the intervention group.

Studies aimed at improving nutritional support by implementing evidence-based algorithms have failed to demonstrate significant positive effects on survival, but the nutritional goals as proposed for the surrogate markers for optimal nutrition were not achieved [5-7]. The lack of findings of clinically relevant effects of nutritional therapy in earlier studies might thus be explained by not attaining adequate provision of energy and protein.

In the present study we analyze the effects of reaching energy provision guided by indirect calorimetry and provision of at least 1.2 g/kg pre-admission body weight. We sought for an effect of optimal nutrition on mortality as outcome parameter.

## Materials and methods

The study was prospectively undertaken in a group of mixed medical-surgical patients in a tertiary university hospital. According to the American Association for Respiratory Care (AARC) guidelines [8], we selected patients who require long-term acute care, patients with a known or suspected nutritional deficit, and subgroups with a nutritional and stress factors that may considerably skew prediction by Harris-Benedict equation. The long-term acute care patients were included if on days three to five (timing of indirect calorimetry) the foreseeable period of artificial nutrition was another five to seven days at least; if age was over 18 years and if it was a first admission to the ICU during the hospital stay. Limiting factors for inclusion were: fraction of inspired oxygen of more than 0.6, air leaks through cuffs and or chest drains, limited availability due to usage of the metabolic monitor or of the two intensivists who performed the measurements, service/repair of defects of the only metabolic monitor available, and withdrawal of treatment because of poor prognosis.

The study was approved by the ethics committee of the VU University Medical Center. The need for informed consent was waived because no additional procedures apart from usual intensive care practice were involved and the data used in this study needed to be collected for clinical purposes. The Dutch legislation does not require informed consent for such clinical protocol-based treatment and data collection, provided that the results are anonymous.

Our nutritional protocol is aimed at early enteral feeding, starting within 24 hours after admission [see Additional data file 1]. The choice for calculating resting energy expenditure (REE) as Harris-Benedict times 1.2 originates from the recommendation by Alexander and colleagues [9] where actual REE are compared with formulas used in the ICU. Also the AARC guidelines uses the Harris-Benedict equation. The 10% extra for activity originates from a study by van Lanschot and colleagues [10] where 24 hour indirect calorimetric measurements were performed to determine total energy expenditure (TEE).

Thus, the energy target is determined by the Harris-Benedict 1984 equation plus 30%, until indirect calorimetry is performed [11]. Indirect calorimetric measurements are performed as part of routine care, usually between day three and five after admission, according to the AARC guidelines [8].

After the measurement, the caloric goal was set at the measured REE plus 10% for activity, and nutrition was adjusted to meet the new caloric goal. Repeated measurements were performed when clinically indicated, according to the AARC guidelines. Caloric provision was tailored towards the latest calorimetric measurement. Protein was provided with a target of 1.2 to 1.5 g/kg pre-admission body weight. According to Dutch guidelines on protein provision, patients with a BMI of more than 30 kg/m^2 ^are corrected for overweight to calculate their protein need; a BMI of 27.5 kg/m^2 ^was used to compute the corresponding weight and required amount of protein/kg/day [12].

To achieve both energy and protein goals we used an algorithm for enteral nutrition that determines the nutritional formula and amount to be given to meet both requirements [13]. The enteral nutritional formulas used are: Nutrison standard^® ^(1000 kcal and 40 g of protein per 1000 ml); Nutrison protein plus^® ^(1250 kcal and 63 g of protein per 1000 ml; both from Numico, Zoetermeer, The Netherlands); and Promote^® ^(1000 kcal and 63 g of protein per 1000 ml; from Abbott Nutrition, Hoofddorp, The Netherlands). Parenteral nutrition during the study period was initially provided by our pharmacy as an all-in-one solution containing 1000 kcal and 47 g of protein per litre, and later a commercially available product was used (Struktokabiven, Fresenius-Kabi A.G., Bad Homburg v.d. H, Germany) containing 1050 kcal and 50 g of protein per litre.

Data from indirect calorimetric measurements have been entered in our data-management system (Metavision^®^, IMD-soft, Tel-Aviv, Israel) since August 2004 and inclusion started from this date. Data retrieval was performed in March 2006. The REE was measured with a calorimeter (Deltatrac™ MBM-100 Metabolic Monitor, Datex-Engstrom Division, Instrumentation Corp. Helsinki, Finland) connected to the ventilator in mechanically ventilated patients. Measurements were performed over a period of 1 to 1.5 hours in resting conditions, after calibration of the device.

For every patient age (years), gender, weight (kg) and height (cm), BMI (kg/m^2^), acute physiology and chronic health evaluation (APACHE) II score, diagnosis group, length of stay in the ICU (ICU-LOS), length of ventilation (LOV), estimated TEE (Harris-Benedict 1984 plus 30%), measured REE from which the TEE was calculated as REE plus 10%, daily energy and protein intake from all sources but oral intake during the period of mechanical ventilation and all blood glucose values during the ICU admission period were recorded. Data for ICU-LOS and data on mortality that could not be extracted from the local ICU database were retrieved from the hospital information system. For every individual patient the probability of death was calculated from the APACHE score, from which the Standardized Mortality Ratio for groups was calculated [14].

For weight and height of the patients we used pre-admission data, retrieved from the pre-assessment outpatient clinic, from earlier measurements taken during admission or from data obtained in other health care settings. Otherwise, the relatives or if possible the patient was asked to provide these data. If these data could not be retrieved, weight was estimated and height was either measured or estimated by one of the two experienced intensivists who performed the indirect calorimetric measurements.

### Nutritional data and calculations

The energy target was set at 90% of the TEE value. Until indirect calorimetry was performed, the daily energy target was calculated with the Harris-Benedict 1984 equation plus 30%. From the day that calorimetric data were available, TEE was defined as measured REE plus 10%, which was then used as energy target. If indirect calorimetry was performed more than once, the new TEE value was used to define the TEE from the moment of measurement. The protein target was defined as 1.2 to 1.5 g of protein/kg pre-admission bodyweight/day. In case of obesity, weight was corrected to a BMI of 27.5 kg/m^2^. Data on caloric and protein intake from artificial nutrition are routinely recorded in our patient data-management system. As our mechanically ventilated patients are fed with artificial nutrition, and oral intake is stimulated after extubation, we used the LOV period for our calculations of energy and protein balances. Energy balance was calculated as energy intake minus energy target, on a daily basis. The protein balance was calculated as total daily intake of protein minus 1.2 g times pre-admission body weight in kg. From the daily energy and protein balances a cumulative balance was calculated for the LOV period and compared with the target values for the entire period of mechanical ventilation. In this way patients could be categorized into four groups according to whether energy and protein goals were reached or not reached.

Determination of adequacy of the glycemic control was performed by calculation of the hyperglycemic index (HGI) in mmol/L per patient during the entire ICU period. The average number of glucose samples per patient in our unit is 6.2 per day. The HGI is defined as the area under the curve above the upper limit of normal (glucose level 6.0 mmol/l) divided by the total ICU-LOS [15].

The outcome variables were death from any cause in the ICU, 28-day mortality and hospital mortality.

### Statistical analysis

Descriptive data are reported as mean and standard deviation, median and range, or as frequency and percentage.

Cox regression analysis with the hospital LOS as time variable, ICU, 28-day and hospital mortality as outcome variables and nutritional goal achieved (yes/no), energy goal achieved (yes/no), and protein goal achieved (yes/no) as independent variables. As gender was found to be a significant effect modifier, data were analysed for males and females separately. All presented hazard ratios (HR) were corrected for weight, height, APACHE II score, diagnosis category, and HGI. SPSS 14 (SPSS Inc., Chicago, IL, USA) was used for statistical analysis. A *P *< 0.05 was considered statistically significant.

## Results

Two hundred and forty-three sequential patients fulfilled the inclusion criteria. Of these, 184 patients were fed exclusively with enteral nutrition, four patients were exclusively fed with parenteral nutrition and 55 patients received enteral and parenteral nutrition during the period of mechanical ventilation. The Harris-Benedict formula prior to the indirect calorimetric measurement underestimated in 13.2% by less than 10%, 70.4% of the estimations was within +/- 10% and in 16.5% overestimated by more than 10%; a bias of +0.9% makes the prediction acceptable for a group. However, the prediction can strongly deviate from the indirect calorimetric value for individual patients with a maximal negative error of 23.8% and maximal positive error of 38.8%. The median period between admission and indirect calorimetry was six days.

According to achievement of the cumulative nutritional goals the patients were placed into one of four groups. Demographic, clinical and nutritional data are presented in Table 1 and Table 2 for males and females separately. Females reached nutritional goals more often than men (34/102; 33.3% vs 25/141; 17.7%).

**Table 1:** Patients' characteristics by nutritional group (male patients)

	E-/P-^1^ (n = 82)	E+/P-^2^ (n = 29)	E-/P+^3^ (n = 5)	E+/P+^4^ (n = 25)	Total (n = 141)
Age (years)					
Mean (SD)	61.67 (15.63)	62.10 (20.30)	55 (14)	62.28 (19.84)	61.63 (17.28)
Median (range)	65 (24–84)	69 (19–91)	46 (44–74)	65 (20–87)	65 (19–91)
					
Height (cm)					
Mean (SD)	178.32 (6.15)	177.38 (7.02)	174.8 (8.30)	175.68 (11.92)	177.53 (7.70)
Median (range)	180 (165–192)	180 (160–190)	176 (165–185)	175 (155–203)	178 (155–203)
					
Weight (kg)					
Mean (SD)	83.46 (14.74)	72.16 (16.30)	84.8 (38.35)	72.16 (16.30)	80.58 (15.97)
Median(range)	80 (54–130)	70 (45–110)	75 (47–130)	70 (45–110)	80 (45–130)
					
BMI (kg/m2)					
Mean (SD)	26.20 (4.15)	25.07 (2.91)	27.92(13.44)	23.6 (6.38)	25.57 (5.00)
Median(range)	26.1 (17.8–40.1)	24.7 (18.5–34)	23.1 (15.2–47.8)	23.1(15.5–45.8)	24.9 (15.2–47.8)
					
APACHE	II				
Mean (SD)	21.87 (8.64)	21.14 (6.85)	16.2 (12.07)	20.04 (6.51)	21.19 (8.09)
Median(range)	20.5 (4–46)	20 (11–38)	11 (5–32)	19 (8–36)	20 (4–46)
					
HGI (mmol/L)^5^					
Mean (SD)	1.331 (0.55)	1.097 (0.55)	1.32 (0.78)	1.076 (0.56)	1.237 (0.57)
Median (range)	1.26 (0.35–2.85)	1.04 (0.30–2.55)	1.10 (0.71–2.66)	0.94 (0.43–2.82)	1.18 (0.30–2.85)
					
LOV^6^					
Mean (SD)	20.65 (23.54)	24 (20.11)	38.4 (45.20)	20.64 (12.86)	21.96 (22.36)
Median (range)	15 (2–181)	18 (4–83)	18 (14–119)	19 (2–48)	16 (2–181)
					
LOS ICU^7^					
Mean (SD)	24.33 (27.65)	26.9 (21.25)	41.6 (46.96)	23.84 (14.74)	25.31 (25.37)
Median (range)	17 (3–209)	21 (4–83)	20 (15–125)	21 (5–64)	19 (3–209)
					
LOS hospital^8^					
Mean (SD)	42.15 (32.83)	46.66 (29.23)	103.6 (111.83)	42.72 (26.32)	45.35 (37.41)
Median (range)	33 (4–210)	41 (6–126)	43 (31–297)	40 (6–95)	36 (4–297)
					
Energy intake (kcal/day)					
Mean (SD)	1627 (368.47)	1883 (341.75)	1936 (518.85)	1853 (446.25)	1730 (398.85)
Median	1624	1957	1994	1894	1771
Range	558–2620	1284–2543	1267–2545	734–2633	558–2633
					
Energy cumulative end balance (kcal)					
Mean	-9149.71	-2177.69	-22967	2336.52	-6169.16
SD	7946.35	4447.88	36552.77	5700.77	10758.06
Median	-6637.5	-1270	-8408	86	-4407
Range	-44504 -733	-15615 +3649	-88277 -4597	-4423 +20989	-88277 +20989
					
Protein intake (g/day)					
Mean (SD)	66.33 (18.74)	82.05 (12.2)	91.84 (28.49)	93.83 (17.03)	75.34 (20.82)
Median (range)	69.48 (12–100)	83.91 (57–111)	100.89 (58–123)	91.75 (66–131)	74.73 (12–131)
					
% days energy goal not reached					
Mean (SD)	55.29 (22.64)	19.05 (13.53)	53.38 (19.81)	15.74 (11.19)	40.76 (26.54)
Median(range)	51.19 (18–100)	17.31 (0–61)	52.94 (29–83)	12.50 (0–50)	35.14 (0–100)
					
% days protein goal not reached					
Mean (SD)	74.71 (21.06)	59.56 (23.77)	40.39 (34.04)	20.09 (12.45)	60.69 (29.22)
Median(range)	73.70 (33–100)	50.00 (22–100)	27.78 (18–100)	20.00 (0–50)	61.54 (0–100)
					
Admission diagnosis (%)					
Trauma	7.3%	6.9%	20%	20%	10%
Sepsis	15.9%	17.2%	-	16%	15.6%
Resp. Insuff	19.5%	27.6%	20%	40%	24.8%
Post-surgical	36.6%	37.9%	60%	20%	34.8%
Neurological	8.5%	10.3%	-	4%	7.8%
Post-resuscit	12.2%	-	-	-	7.1%
Total	100%	100%	100%	100%	100%
					
Mortality (%)					
ICU	15.9%	17.2%	20%	24%	17.7%
28-day	17.1%	13.8%	0%	24%	17%
Hosp	30.5%	27.6%	40%	36%	31%
					
SMR^9^					
	0.39	0.47	0.80	0.80	0.47

^1^E-/P- = energy and protein targets not reached

^2^E+/P- = energy target reached and protein target not reached

^3^E-/P+ = energy target not reached and protein target reached

^4^E+/P+ = energy and protein targets reached

^5^HGI = hyperglycemic index, see methods

^6^LOV = length of ventilation

^7^LOS ICU = length of stay at intensive care unit

^8^LOS hospital = length of stay in hospital

^9^SMR = standardized mortality rate; observed mortality/predicted mortality

APACHE = acute physiology and chronic health evaluation; SD = standard deviation.

**Table 2:** Patients' characteristics by nutritional group (female patients)

	E-/P-^1^ (n = 32)	E+/P-^2^ (n = 35)	E-/P+^3^ (n = 1)	E+/P+^4^ (n = 34)	Total (n = 102)
Age (years)					
Mean (SD)	62.59 (21.90)	67.54 (12.86)	78 (-)	63.47 (16.26)	64.74 (17.20)
Median (range)	72.5 (19–88)	71 (26–84)	78	68 (20–86)	70 (19–88)
					
Height (cm)					
Mean (SD)	169 (6.89)	164.8 (6.60)	173 (-)	163.24 (5.73)	165.68 (6.80)
Median (range)	170 (152–180)	165(152–178)	173	163.5 (150–175)	165 (150–180)
					
Weight (kg)					
Mean (SD)	68.75 (12.88)	68.8 (13.81)	58 (-)	59.47 (9.68)	65.57 (12.87)
Median(range)	67.5 (36–101)	65 (50–112)	58	59.5 (40–78)	65 (36–112)
					
BMI (kg/m2)					
Mean (SD)	24.08 (4.31)	25.34 (4.85)	19.4(-)	22.28 (3.14)	23.87 (4.32)
Median(range)	23.45(12.5–34.6)	24.7 (17.3–39.7)	19.4	22.25(15.6–30.1)	23.55 (12.5–39.7)
					
APACHE	II				
Mean (SD)	19.62 (7.17)	21.31 (7.43)	20 (-)	21.56 (7.32)	20.85 (7.25)
Median(range)	18.5 (7–36)	18 (8–35)	20	21 (9–46)	20 (7–46)
					
HGI (mmol/L)^5^					
Mean (SD)	1.014 (0.44)	1.259 (0.51)	1.48 (-)	1.048 (0.41)	1.115 (0.46)
Median (range)	0.95 (0.38–2.51)	1.15 (0.55–2.54)	1.48	1.02 (0.45–2.12)	1.07 (0.38–2.54)
					
LOV^6^					
Mean (SD)	13.66 (10.66)	28.66 (20.43)	11 (-)	28.82 (21.70)	23.83 (19.50)
Median (range)	12 (3–59)	22 (7–91)	11	22 (3–102)	18 (3–102)
					
LOS ICU^7^					
Mean (SD)	15.81 (10.72)	31.31 (20.50)	17 (-)	31.44 (22.72)	26.35 (19.97)
Median (range)	14.5 (3–59)	24 (9–91)	17	25 (4–110)	21 (3–110)
					
LOS hospital^8^					
Mean (SD)	37.47 (34.38)	50.83 (36.49)	43 (-)	55.38 (34.70)	48.08 (35.51)
Median (range)	26 (4–176)	43 (10–181)	43	51 (9–162)	40 (4–181)
					
Energy intake (kcal/day)					
Mean (SD)	1364 (355.04)	1624 (227.02)	1673 (-)	1603 (269.83)	1536 (298.96)
Median	1413	1601	1673	1592	1547
Range	65–1992	1170–2046		818–209265-2092	
					
Energy cumulative end balance (kcal)					
Mean	-5467.66	-1335.34	-3955	2765.82	-1290.38
SD	3909.27	3189.68	(-)	5349.70	348.33
Median	-5049	-813	-3955	1549	-1212.5
Range	-20533 -836	-10630 +3390		-6057 +18354	-20533 +18354
					
Protein intake (g/day)					
Mean (SD)	53.79 (18.92)	65.97 (16.33)	75.36 (-)	80.34 (11.83)	67.03 (19.02)
Median (range)	55.82 (0–89)	69.52 (4–84)	75.36	79.79 (56–100)	69.55 (0–100)
					
% days energy goal not reached					
Mean (SD)	58.85 (21.61)	21.77 (8.66)	45.45 (-)	17.00 (11.50)	32.05 (23.54)
Median(range)	58.57 (29–100)	21.43 (7–44)	45.45 (45–45)	16.67 (0–44)	25.41 (0–100)
					
% days protein goal not reached					
Mean (SD)	72.42 (25.77)	63.83 (29.05)	45.45 (-)	20.96 (11.60)	52.06 (32.20)
Median(range)	78.18 (22–100)	64.71 (14–100)	45.45 (45–45)	17.31 (0–50)	45.45 (0–100
					
Admission diagnosis (%)					
Trauma	9.4%	5.7%	-	2.9%	5.9%
Sepsis	15.6%	8.6%	-	11.8%	11.8%
Resp. Insuff	25.0%	34.3%	-	41.2%	33.3%
Post-surgical	31.2%	34.3%	-	23.5%	29.4%
Neurological	9.4%	8.6%	100%	14.7%	11.8%
Post-resuscit	9.4%	8.6%	-	5.9%	7.8%
Total	100%	100%	100%	100%	100%
					
Mortality (%)					
ICU	25%	25.7%	0%	11.8%	20.6%
28-day	28.1%	17.6%	0.%	5.9%	16.7%
Hosp	31.2%	34.3%	0%	26.5%	30.4%
					
SMR					
	0.74	0.74	-	0.31	0.58

^1^E-/P- = energy and protein targets not reached

^2^E+/P- = energy target reached and protein target not reached

^3^E-/P+ = energy target not reached and protein target reached

^4^E+/P+ = energy and protein targets reached

^5^HGI = hyperglycemic index, see methods

^6^LOV = length of ventilation

^7^LOS ICU = length of stay at intensive care unit

^8^LOS hospital = length of stay in hospital

^9^SMR = standardized mortality rate; observed mortality/predicted mortality

APACHE = acute physiology and chronic health evaluation; SD = standard deviation.

The results of the statistical analysis are presented in Table 3. Cox regression analysis showed no significant effects of attaining nutritional goals on mortality in men.

**Table 3:** Hazard ratios, confidence intervals and *P *values for mortality in the female part of the population between groups according to different combinations of energy and protein goals reached.

	ICU mortality	28 day mortality	Hospital mortality
**Males**			
E+/P+ versus E-/P-^a^ (n = 25 vs 82)	1.602; 0.464–5.524; *P *= 0.456	1.060; 0.320–3.514; *P *= 0.924	1.106; 0.413–2.961; *P *= 0.841
E+/P- versus E-/P-^b^ (n = 29 vs 82)	1.161; 0.402–3.355; *P *= 0.783	0.721; 0.233–2.231; *P *= 0.570	0.838; 0.367–1.916; *P *= 0.676
P+/(E+,E-) versus P-/(E+,E-)^c^ (n = 30 vs 111)	1.095; 0.384–3.116; *P *= 0.866	0.821; 0.273–2.469; *P *= 0.726	0.939; 0.399–2.213; *P *= 0.886
E+/(P+,P-) versus E-/(P+,P-)^d^ (n = 54 vs 87)	1.146; 0.478–2.750; *P *= 0.760	0.966; 0.402–2.320; *P *= 0.938	1.017; 0.519–1.992; *P *= 0.960
**Females**			
E+/P+ versus E-/P- (n = 34 vs 32)	0.199; 0.048–0.831; *P *= 0.027	0.079; 0.013–0.467; *P *= 0.005	0.328; 0.113–0.952; *P *= 0.04
E+/P- versus E-/P- (n = 35 vs 32)	0.341; 0.102–1.141; *P *= 0.081	0.120; 0.027–0.528; *P *= 0.005	0.318;0.107–0.945; *P *= 0.039
P+/(E+,E-) versus P-/(E+,E-) (n = 35 vs 67)	0.295; 0.090–0.964; *P *= 0.043	0.176; 0.037–0.838; *P *= 0.029	0.405; 0.168–0.977; *P *= 0.044
E+/(P+,P-) versus E-/(P+,P-) (n = 69 vs 33)	0.332; 0.116–0.949; *P *= 0.040	0.137; 0.041–0.461; *P *= 0.001	0.351; 0.145–0.847; *P *= 0.020

^a ^E+/P+ versus E-/P- = both cumulative energy and protein goal reached versus both energy and protein goal not reached

^b ^E+/P- versus E-/P- = cumulative energy goal reached versus energy goal not reached, for both groups protein goal not reached

^c ^P+/(E+,E-) versus P-/(E+,E-) = cumulative protein goal reached versus protein goal not reached, irrespective of attaining energy goal

^d ^E+/(P+,P-) versus E-/(P+,P-) = cumulative energy goal reached versus energy goal not reached, irrespective of attaining protein goal

**Figure 1:**
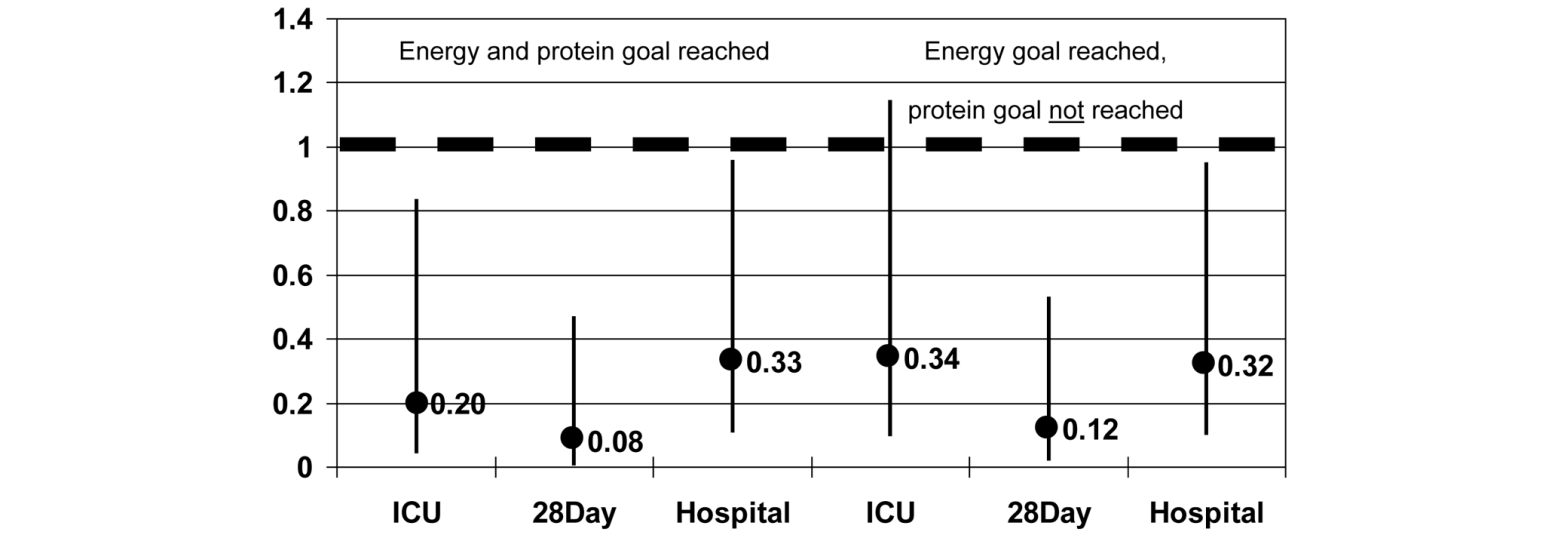
Hazard ratios for women according to energy goal reached and protein goal reached or not. ICU = intensive care unit.

For the female part of the population, the HRs for ICU, 28-day and hospital mortality were significantly lower for the group that reached both energy and protein goals compared with the group that did not reach both goals. The strongest effects were seen on 28-day mortality (HR = 0.079; confidence interval (CI) = 0.013 to 0.467; *P *= 0.005). The effects of reaching both energy and protein goals are more obvious than when only the energy target is reached (Figure 1). In the latter case, the HR for ICU mortality did not reach significance. The HRs for hospital mortality, however, are equivalent between these two groups.

Table 3 also shows the results for comparison of the groups that reached the protein goal or not, irrespective of the energy goal, and results of reaching the energy goal or not, irrespective of the protein goal. Analysis of the Standardized Mortality Ratio per nutritional goals group and per gender showed a low predicted/observed mortality for women who reached both the energy and protein goal, but for men this effect was absent.

## Discussion

Reaching nutritional goals, in this study defined as energy delivery with a minimum of 90% of the measured REE plus 10% and protein provision of at least 1.2 g/kg pre-admission body weight during the period of mechanical ventilation, results in an 80% decreased chance of dying in the ICU and a 92% decreased 28-day mortality, while hospital mortality is 67% lower when compared with patients who do not reach the above mentioned nutritional goals. These effects only occur in the female part of the ICU population. In men, no statistically significant effects of nutrition on outcome could be detected.

Reaching only the energy target and not attaining 1.2 g protein/day in females results in less favorable outcomes than when both energy and protein goals are reached. The chance of dying in the ICU is not affected by reaching only the energy target but there is still a decreased chance of dying of 88% at 28 days and a 68% decrease of hospital mortality.

Women have a lower body weight as a group and thus less energy expenditure than men. As administration of the volume of enteral nutrition formulas is a limiting factor early in the course of nutritional therapy, women are more likely to reach their nutritional goals.

The energy deficit occurs especially in the first days after admission, when targeted volume cannot be administered due to retention, slow increase of nutritional volume towards the targeted volume, hemodynamic instability and diagnostic and therapeutic interventions.

Recently, Pichard and colleagues [16] have demonstrated that provision of more than 1500 kcal/day in the first three days of admission besides parenteral glucose reduces ICU mortality and hospital mortality. Early provision of energy diminishes the cumulative caloric deficit.

To our knowledge, our study is the first one in which beneficial effects of both energy and protein provision on mortality in critically ill patients have been demonstrated.

We can only speculate to explain the differences between gender that we found. No data on body composition changes during ICU or hospital stay are available, and we did not perform nitrogen balances and endocrine investigations towards gender differences. A possible explanation for the difference in effect of nutritional therapy between men and women might be that an absolute minimum of protein content in the body is critical for survival. Beyond this hypothesized protein threshold, loss of organ function and failing immune status will predispose to death. If this was true, males have an advantage in nutritional reserve, because they are heavier and also have a more favorable proportionality between fat and protein, with larger relative protein stores [17]. Thus, females have a disadvantage because they will reach this presumed minimum protein threshold in a shorter period of time during catabolism. Adequate nutrition aims to protect the body composition and slows down catabolism. With the smaller reserve that females have, the effects of nutrition will be more obvious. This is in line with our findings. The beneficial effects of optimal nutrition are also reflected by the low standardized mortality ratio in females who reach their nutritional goals, while this effect is not seen in the male group which can be expected because in the statistical analysis no effect of optimal nutrition could be demonstrated. The standardized mortality ratio rests on an accurate APACHE score, which can be subject to errors [18]

How can we explain that others have not found effects of nutrition on mortality and why has female gender not been recognized as an important factor? Our study included a relatively large number of patients, of whom 42% (n = 102) were women. We used the Harris-Benedict equation until indirect calorimetry was performed and used the measured energy expenditure as target for energy provision thereafter: adequacy of energy and protein provision was strictly defined and the entire period of ventilation was taken as the study period. Furthermore, we calculated a HGI for every patient to assess glycemic control. In the studies by Villet and colleagues [2] and Dvir and colleagues [3] the relative small number of patients (48 and 50, respectively) and the predominance of males in both studies (30 and 33, respectively) may have blurred the effects of gender differences. Both studies were looking for the negative effects of energy deficits during ICU stay, and did not focus on the effects of adequate nutrition in terms of both energy and protein delivery. In the study by Villet and colleagues the energy target was set at measured REE plus 30% (69% of patients underwent indirect calorimetry) or calculated as 30 kcal/kg/day. Dvir and colleagues performed daily indirect calorimetric measurements, and used the measured REE as target for the caloric intake. No data on protein provision or glycemic control are given for both studies. In a study by Barr and colleagues, the outcome parameter for adequacy of nutritional support was energy provision on day four of nutritional support. Caloric target estimates were determined by using the Harris-Benedict equation. Remarkably, the percentage of the targeted caloric provision on day four decreased from 73% in the preimplementation group to 67% after implementation of the protocol, and the lack of effect on mortality might thus be explained by inadequate provision of energy. No information on protein provision or glycemic control was provided [5]. In the ACCEPT study, the provision of energy after implementation of algorithms to improve caloric intake was most probably insufficient compared with the energy target used: in the intervention group the provision of calories was 1264 kcal per patient day, compared with 998 kcal in the control group. The amount of protein delivered was 0.41 g/kg/day, compared with 0.37 g/kg/day in the control group [6]. In the study by Doig and colleagues [7], also implementing evidence-based feeding guidelines, no statistically different amounts of energy and protein were delivered to the intervention group compared with the control group (1241 kcal/day and 1065 kcal/day, respectively; and 50.1 g/day and 44.2 g/day, respectively) and thus much lower than the targets that we set for energy and protein as considered minimal in our study [6]. Also in these studies, no data on glycemic control were provided.

Thus, it is plausible that differences in study designs, numbers of patients included, different definitions for nutritional goals and analyses on group level instead of analyses on the level of individual patients account for finding different effects of nutrition on mortality.

Our study has limitations. It is an observational study. Neither body composition was established nor were nitrogen balances performed, so that the hypothesized correlation between net protein loss and mortality could not be substantiated. As in similar studies, the pre-admission weight was not accurately known for all patients. Although, in the statistical analysis, we corrected for weight, height, APACHE-II, diagnosis group and glycemic control, it is possible that other factors may have influenced mortality. Although the hypothesis of optimal nutrition does not take gender into consideration, we could demonstrate only an effect on mortality in women. Furthermore, the recommendations for the amounts of energy and protein provision in critically ill patients originate from only a limited number of studies and might prove to be insufficiently tailored towards the individual needs in such a diverse population [19-22].

## Conclusions

In conclusion, the main finding of our study is that reaching both an energy goal guided by indirect calorimetry and provision of protein in an amount of at least 1.2 g/kg pre-admission body weight during the period of artificial nutrition while mechanically ventilated, reduces ICU, 28-day and hospital mortality in the female part of the population. The favorable effect in women on ICU mortality could not be demonstrated for those who reached the energy goal but failed to attain 1.2 g of protein/kg/day. For males no beneficial effects on mortality could be shown of reaching these nutritional targets during the period of artificial ventilation.

Although our findings must be confirmed by others, we argue that the observed beneficial effects of nutrition in females are so pronounced, that an ultimate effort should be made to secure adequate provision of both energy and protein. Further research is needed to elucidate the underlying mechanisms to explain the relation between nutrition, gender and mortality in ICU patients.

## Key messages

• Optimal nutrition for intensive care patients can be defined as provision of energy as actually used and protein in an amount of 1.2 to 1.5 g/kg pre-illness body weight/day.

• So far the goals of optimal nutrition were surrogate endpoints; this study shows that for long-term acute care of female patients, optimal nutrition affects clinically relevant outcomes.

• Female patients who reach their energy and protein goals have significantly lower ICU, 28 day- and hospital mortality compared with those who do not reach these goals.

• In the long-term acute care of female patients reaching both energy and protein goals is more advantageous than reaching only the energy goal: in the latter case ICU mortality is not affected and the effect on 28-day mortality is less obvious, which suggests that the beneficial effect of also reaching the protein goal is especially important in the early phase of critical illness.

• In the present study, beneficial effects of optimal nutrition could not be demonstrated in the male part of our population.

## Abbreviations

AARC: American Association for Respiratory Care; APACHE: acute physiology and chronic health evaluation; CI: confidence interval; HGI: hyperglycemic index; HR: hazard ratio; LOS: length of stay; LOV: length of ventilation; REE: resting energy expenditure; TEE: total energy expenditure.

## Competing interests

The authors declare that they have no competing interests.

## Authors' contributions

The study was designed by all authors. Caloric measurements were performed by RS and AB. Data retrieval and statistical analyses were performed by RK and PW. HS and RS defined optimal nutrition. All authors were involved in the several stages of writing the manuscript. RS and PW had full access to all of the data in the study and take responsibility for the integrity of the data and the accuracy of the data analysis.

## Supplementary Material

Additional file 1 A Word file describing the nutrition, sedation and weaning protocol of the ICU. Click here for file
