# Supplementary material for: Optimal nutrition during the period of mechanical ventilation decreases mortality in critically ill, long-term acute female patients: a prospective observational cohort study
Source: Crit Care. 2009 Aug 11;13(4):R132. doi: 10.1186/cc7993 (PMC2750190; doi:10.1186/cc7993)
Supplement: Additional file 1 — A Word file describing the nutrition, sedation and weaning protocol of the ICU. [file cc7993-S1.doc]

Additional data file 1:

We start as early as possible with nutritional therapy, after hemodynamics are stable. The route of administration is preferably via the intestinal tract. We allow gastric residuals of 250 ml/6 hours. If residuals surpass this amount we administer erythromycin 1-3 mg/kg body weight for 2 days. In case of no effect the nutrition is administered post pyloric. Parenteral nutrition is provided only when the gut fails (fistulas, short bowel, obstruction) and is not given as parenteral suppletion to inadequate amounts of enteral nutrition in the early phase of nutritional therapy. Target nutrition for both energy and protein is defined by an algorithm in our patient data management system that has been published and is referenced in the text (13). In case of nutritional deficits and the use of parenteral nutrition we supply glutamine for 9 days.

Sedation protocol: we adhere to the guideline “sedation and analgesia” of the Dutch Society for Intensive Care (http://www.nvic.nl/richtlijnen_geaccordeerd.php.) Starting point for this guideline is patient comfort, allowance of intensive therapy and guarantee of sufficient retrograde amnesia. On a regular basis the effect of the sedatives must be evaluated, through the Ramsey score. The dosage should be adapted to this score and the indication for sedation should be evaluated on a daily basis. Propofol is used for short term sedation, midazolam is used for prolonged sedation. Morphine is used as a first choice analgesic; fentanyl is recommended in critically ill patients with hemodynamic instability.

Weaning protocol: we adhere to the guideline “weaning from the ventilator” of the Dutch Society for Intensive Care (http://www.nvic.nl/richtlijnen_geaccordeerd.php) Key points in this guideline are: weaning from the ventilator is as effective with pressure support or T-piece weaning and these are superior tot SIMV; weaning through a protocol shortens the period of mechanical ventilation; weaning trial of 30 minutes results in the same success rate than a weaning trial of 120 minutes.
